# Conceptual model to inform *Legionella*–amoebae control, including the roles of extracellular vesicles in engineered water system infections

**DOI:** 10.3389/fcimb.2023.1200478

**Published:** 2023-05-18

**Authors:** Nicholas John Ashbolt

**Affiliations:** Faculty of Science and Engineering, Southern Cross University, Lismore, NSW, Australia

**Keywords:** engineered water systems, environmental persistence, disinfection resistance, QMRA, monitoring

## Abstract

Extracellular vesicles (EVs or exosomes) are well described for bacterial pathogens associated with our gastrointestinal system, and more recently as a novel mechanism for environmental persistence, dissemination and infection for human enteric viruses. However, the roles played by EVs in the ancient arms race that continues between amoebae and one of their prey, *Legionella pneumophila*, is poorly understood. At best we know of intracellular vesicles of amoebae containing a mix of bacterial prey species, which also provides an enhanced niche for bacteriophage infection/spread. Free-living amoeba-associated pathogens have recently been recognized to have enhanced resistance to disinfection and environmental stressors, adding to previously understood (but for relatively few species of) bacteria sequestered within amoebal cysts. However, the focus of the current work is to review the likely impacts of large numbers of respiratory-sized EVs containing numerous *L. pneumophila* cells studied in pure and biofilm systems with mixed prey species. These encapsulated pathogens are orders of magnitude more resistant to disinfection than free cells, and our engineered systems with residual disinfectants could promote evolution of resistance (including AMR), enhanced virulence and EV release. All these are key features for evolution within a dead-end human pathogen post lung infection. Traditional single-hit pathogen infection models used to estimate the probability of infection/disease and critical environmental concentrations *via* quantitative microbial risk assessments may also need to change. In short, recognizing that EV-packaged cells are highly virulent units for transmission of legionellae, which may also modulate/avoid human host immune responses. Key data gaps are raised and a previous conceptual model expanded upon to clarify where biofilm EVs could play a role promoting risk as well as inform a more wholistic management program to proactively control legionellosis.

## 1 Introduction

Water-based (saprozoic) pathogens cost the US over $2.39 billion per year (approximately $7m per head of population), some ten-fold the cost of gastrointestinal waterborne pathogens (Collier et al., 2021). These saprozoic pathogens predominantly grow within microeukaryotes, such as free-living amoebae and their analogues, macrophages, differentiating them from saprotrophic microorganisms that live off dead and decaying matter. In Europe, legionellosis (largely caused by three species of saprozoic *Legionella* from drinking water) was identified as the fifth most significant contributor to disability-adjusted life years (DALYs) from 31 selected diseases, following influenza, tuberculosis, human immunodeficiency virus (HIV) infection/AIDS and invasive pneumococcal disease (Cassini et al., 2019). However, unlike the top four infectious diseases, legionellosis is not spread person-to-person, but largely infect humans from water/moist soil/mulch habitats *via* aerosols to lung macrophages and alveolar epithelial cells (Ashbolt, 2015) – placing management in multiple hands, given the various jurisdictions addressing water provision (treatment, distribution and premise) and its other environmental exposures. Also, our aging population and various increasing vulnerable groups to these opportunistic pathogens are escalating the disease burden from water-based pathogens globally.

In the general absence of regulations requiring saprozoic pathogen monitoring near points of potential exposures (Bartram et al., 2007; CDC, 2017; HSE, 2017; Gamage et al., 2022), along with most cases being sporadic and rarely followed up (Abu Khweek and Amer, 2018; Clopper et al., 2021), there is significant under reporting of the true impact from these pathogens (Burillo et al., 2017; Vermeulen et al., 2020). What we know most is from Legionnaires’ Disease outbreaks from cooling tower aerosols, ornamental fountains and within hospitals, but these only account for some 15% of all cases, the bulk of the remainder being sporadic within the community, largely thought to be from premise plumbing systems (Falkinham et al., 2015; NAS, 2020).

Many saprozoic pathogens are transmitted to humans *via* aerosols, typically following distal disinfection processes (e.g., residual chlorine in drinking water, point-of-use UV irradiation and environmental desiccation). However, recent work has shown that extracellular vesicles (EVs) from amoebae, along with their trophozoites and cysts provide orders of magnitude resistance to disinfection for internalized pathogens compared to the freely suspended bacteria and human viruses (Storey et al., 2004; Folkins et al., 2020; Martin et al., 2020; Dey et al., 2022). From a control point of view this is important, as current guidance/regulation removal is only based on disinfection performance of the more readily inactivated freely suspended. Also relevant is *L. pneumophila*’s dual life phases (replicative & transmissive), with replicative cells (typical form used in disinfection studies) repressing, while the transmissive form induces virulence, motility and most importantly for persistence, various stress factors *via* activity of RNA-binding proteins, including CsrA and its thiamine pyrophosphate riboswitch (Sahr et al., 2017). Overall, this begs the question, has *L. pneumophila*, like other amoeba-resisting pathogens (Schmitz-Esser et al., 2010), evolved not only to grow and control host cells (using a large fraction of its genome to do so [(Burstein et al., 2016)]), but is it also adapted to persist within EVs or even generate host EVs to extend its transmissive phase? Recent transcriptomics is helping us to understand the differentially expressed *Legionella* genes within their infected amoebal hosts (Quan et al., 2020; Chauhan et al., 2023). Such techniques have yet to be applied to EVs production and within these vesicles.

Overall, identifying amoeba-associated pathogens may not only identify amplification niches in engineered systems (Thomas and Ashbolt, 2011; Thomas et al., 2014), but also pathogens more likely to be protected and deliver in an infectious dose (Shaheen and Ashbolt, 2018). No such routine amoeba-targeted monitoring is undertaken today but may well provide the proactive approach sought in water safety plans (WSPs) that are used around the world to manage waterborne enteric pathogens (WHO, 2017), but are in their infancy in being adapted to provide safe water for the management of saprozoic pathogens (Papadakis et al., 2018; De Giglio et al., 2021). Hence, the goal of this article is to summarize what is understood about the biology of *L. pneumophila* relevant to its *in-situ* biofilm hosts with the purpose of identifying promising targets to manage this critical saprozoic pathogen of engineered water systems.

### 1.1 Monitoring and management options for *L. pneumophila*

Current culture-based methods for known saprozoic bacterial pathogens (*Legionella* spp., nontuberculous mycobacteria etc.) take 10-days to weeks for confirmation (e.g., (ISO 11731, 2017; Pfaller et al., 2021)) – far too long for a timely response. Further, cells of these pathogens may largely be present in viable but non-culturable (VBNC) states (hence missed by traditional culture methods) yet still infectious to humans (Dietersdorfer et al., 2018). Therefore, specialized amoeba co-culture is recommended for resuscitation (García et al., 2007; Dey et al., 2019a; Dey et al., 2019b; Dey et al., 2020). While still disputed by some, molecular methods are preferred for the management of water systems, such as qPCR (Lee et al., 2011). However, determining the fraction of infectious cells by qPCR is still problematic, given uncertainties in infectivity status and the variable residual chlorine levels likely present in piped water systems (Casini et al., 2018; Donohue et al., 2019; Donohue, 2021). Flow cytometry using immunocapture (Füchslin et al., 2010) or with cell-sorting in combination with qPCR shows promise to also identify VBNC cells, particularly those missed by conventional culture (e.g., ISO 11731:2017-05 pre-treatment procedure) but capable of infecting host cells (Nisar et al., 2023).

Nonetheless, live or dead, high concentrations (in excess of 10^3^ cells/100 mL, (Hamilton et al., 2021)) in distal parts of water delivery systems infer growth in the system that needs to be addressed, else risk exposure to infectious aerosols at some stage. The major amplification site for *L. pneumophila* in water systems is within biofilm amoebae (NAS, 2020). Therefore, herein a prior conceptual model for legionellae growth within pipe biofilms (Shaheen and Ashbolt, 2021) is expanded upon to include EV and possible amoeba monitoring targets and identifies research gaps. Overall, targeting hosts prior to rapid, explosive growth of legionellae is hypothesized as a useful target within a system of checks (flushing, temp <25 or >50°C, disinfectant residual etc.) for a proactive early warning water management system.

## 2 Primary role of free-living amoebae supporting problematic *L. pneumophila* growth

### 2.1 Protist hosts for explosive growth of *L. pneumophila*

The primary hosts for *Legionella* within biofilms are various free-living protozoa, principally free-living amoebae (FLA) (Thomas et al., 2010) and ciliates (Tsao et al., 2019). While ciliates may excrete viable legionellae within fecal pellets for subsequent re-ingestion (Hojo et al., 2012; Berk and Garduño, 2013), *L. pneumophila* released following their lysis of amoebal trophozoites are more likely to lead to explosive growth cycles of phagocytosis-growth-release (Shaheen et al., 2019), given several hundred of cells that can grow per amoeba (Buse and Ashbolt, 2012).

A recent review of FLA is provided by Scheid (2019), in which he describes their diversity and the predatory heterotrophic feeding by trophozoites on biofilm microbiota and extracellular polymeric substances. Indeed, FLA are ubiquitous and instrumental to both biofilm formation and ecological successions. Important amoebal hosts supporting *Legionella* and other amoeba-resisting bacterial (ARB) pathogens include members of *Acanthamoeba*, *Naegleria, Vermamoeba* and *Willaertia* (Thomas et al., 2010). Although the term FLA has no relevance to amoebal taxonomy or phylogeny, it does separate them from the parasitic intestinal amoebae (e.g., *Entamoeba histolytica*) that have significantly reduced genomes due to their host’s providing most needs (Shabardina et al., 2018). Critical to FLA success and dispersal (air, water, soils & by wildlife) is the environmentally robust cyst form, that may also encase/protect beneficial and pathogenic bacteria and their viruses (Thomas and Greub, 2010; Schulz et al., 2020; Shi et al., 2021), many of whom may carry virus-encoded auxiliary metabolic genes (vAMGs) (Yuan and Ju, 2023).

Pertinent to engineered water systems has been the increasing recognition of the importance of suspended flocs/particulates in amoebal planktonic dynamics (Anderson, 2018). While not considered biofilms of fixed surfaces, these planktonic niches may represent sloughed biofilm material or biofilm-like growth on suspended particulates that contribute to microbial activity in piped water systems (Liu et al., 2016) and ultimately the bulk of internalized pathogens transmitted *via* aerosols (Hamilton et al., 2018; Shaheen and Ashbolt, 2021).

### 2.2 Extracellular vesicles of FLA

**Figure 1:**
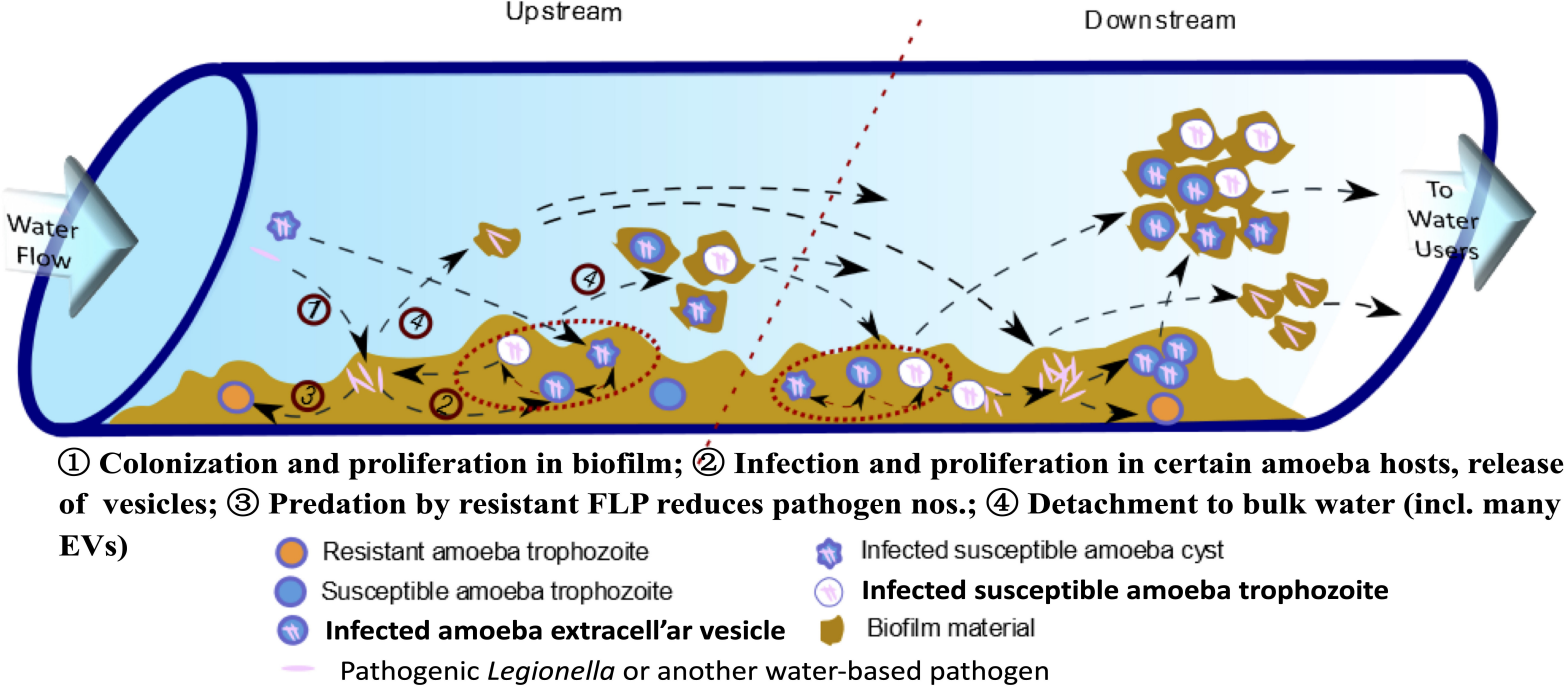
Conceptual model for explosive growth of *Legionella* in engineered water system biofilms. (Adapted from Shaheen et al. (2019), FLP - free-living protozoa [primarily amoebae and ciliates] grow on biofilm constituents, ultimately limited to feeding on less-preferred amoeba-resisting bacterial, such as *L. pneumophila*, leading to explosive growth and release in extracellular amoebal vesicles [EVs] that may enhance downstream amoebal predation over less preferred free bacteria – so increasing proliferation of pathogens.).

Nearly all living cells may excrete EVs, which are described based on their site of origin (i.e., exosomes, ectosomes, cytonemes & nanotubes) (Vermeulen et al., 2020.). However, only recently have EVs been recognized as participating in cell-to-cell communication processes, including between predator and prey (de Souza and Barrias, 2020), and carry specific virulence factors (Cruz Camacho et al., 2023) with known immunomodulatory properties (Costa et al., 2021). Exosomes are EVs that originate from the endocytic pathway of a cell (de Souza and Barrias, 2020; Cruz Camacho et al., 2023) and FLA-EVs are of focus here because of their likely multiple connections across the life cycle of *L. pneumophila* in biofilms of engineered environments (**Figure 1**).

Various drinking water associated FLA have been shown to generate EVs containing tens to hundreds of *L. pneumophila* cells within the respiratory range (< 10 µm dia.) (Shaheen and Ashbolt, 2018). These EVs may provide packages of pathogens directly to the alveoli of our lungs, potentially influencing current quantitative microbial risk assessment (QMRA) models of *Legionella* risks and specifically the dose-response relationship (Hamilton et al., 2019). Hypothesized in this article are the environmental queues that may not only influence EV production but also their role leading to explosive growth of legionellae in their native biofilms (**Figure 1**).

While speculative for FLA, in bacterial predators, such as *Myxococcus xanthus*, outer membrane vesicles (OMVs) appear to specifically carry a ‘personalized’ subset of proteins that only in-part are dictated by the predator’s genome (Zwarycz et al., 2020). In other words, Zwarycz et al. (2020) hypothesize that the non-genome-derived proteome fraction is there to target its prey. Hence, is *L. pneumophila* really the predator not amoebae, utilizing a large fraction of its genome to not only manipulate the hosts into providing a growth niche in the form of *Legionella*-containing vacuoles (LCV) but potentially also EVs production to increase its persistence/transmission? Furthermore, as seen with other EVs, those containing legionellae may also modulate/avoid human host immune responses (e.g., triggered by phosphatidylserine lipids on the surface of EVs (Cole and Nizet, 2016)).

In bacteria, outer membrane vesicles (OMVs) have also been shown to provide a defense mechanism to phage infection (Reyes-Robles et al., 2018). Hence, next genomic then viruses specific to FLA are discussed and how they may impact on the evolution of these important hosts and ARB.

### 2.3 Genomic implications for *L. pneumophila* within engineered systems

As with their FLA hosts suited to the diversity of conditions in environmental biofilms, ARB maintain a larger genome than their relatives – so contradicting the expected genome reduction theory accepted for most intracellular pathogens (Moliner et al., 2010). This is likely due to the importance of gene transfer of pathogenicity factors, and the near 800 million year old arms-race between these predators and prey (Dupont et al., 2011; Shames, 2023) along with the role played by their respective viruses (Kaján et al., 2020). Of particular focus in this article is the remodeling of the *Legionella*-containing vesicle (LCV), with legionellae acquiring effector protein genes, such as Sar1/CopII that impact early secretory vesicle production genes (Robinson and Roy, 2006). As introduced in **Figure 1**, it is unknown how ARB may influence the release of EVs to enhance predator infection downstream, and provide a hot-spot for horizontal gene transfer (HGT). Recent work has pointed to FLA preferentially preying on non-ARB free bacteria over *L. pneumophila* (Shaheen and Ashbolt, 2021). What is unknown, but hypothesized here is that legionellae within EVs may not only avoid environmental stressors, but also overcome negative selective feeding on *L. pneumophila* by FLA. Indeed, are EVs preferential prey for FLA and an enhanced mechanism for prey to counter predators’ sensing of preferred food?

The role played by quorum sensing molecules (such as LAI-1 [3-hydroxypentadecane-4-one]) involved in legionellae changing from a reproductive to a transmissive phase (Schell et al., 2016), could also be part of the evolutionary arms race between predator and prey (Simon et al., 2015). It is currently unknown if LAI-like molecules are activated within EVs, as known to occur within trophozoites. Other potential changes within trophozoites and EVs that HGT may also impact could include environmental antimicrobial resistance (AMR) (Mortensen et al., 2021). AMR has generally not been viewed as important with pathogens that do not spread zoonotically or by person-to-person (such as with *L. pneumophila*). However, AMR within amoebal ecosystems increasingly impacted by AMR (e.g., cooling towers or irrigation systems receiving AMR-laden wastewater) could present a ‘perfect storm’ for enhance AMR within *L. pneumophila*-FLA ecosystems.

### 2.4 FLA evolution and their nucleocytoplasmic large DNA viruses

FLA evolved some 800-850 My ago (Cavalier-Smith, 2009), but have and continue to have exchange with bacteria/archaea and related viruses. A particular feature of FLA are their nucleocytoplasmic large DNA viruses (NCLDV), first described in 2003 (Abrahao et al., 2014) as *Acanthamoeba polyphaga* mimivirus (APMV) (now in the genus *Mimivirus*). In a recent whole genome analysis of *A. polyphaga* various NCLDVs (*Marseillevirus, Mimivirus, Mollivirus, Pandoravirus*, *Pithovirus* and a yet to be described family) were identified - inferring that NCLDV may have been domesticated during the evolution of amoebae (Schulz et al., 2020). Hence, though considered rather unusual, NCLDVs are not just a result of recent horizontal gene transfer (HGT) but in fact are associated with most major eukaryotic lineages, so impacting all ecosystems on earth (Schulz et al., 2020).

Another feature, potentially relevant to *L. pneumophila*’s human infectivity, has been described for human enteric viruses. A good exemplar is seen with human *Norovirus*, which change commensal gut bacteria’s EV production, likely impacting human host responses to infection (Mosby et al., 2022). While speculative for human infection by amoeba-legionellae material, it is intriguing that various enveloped and non-enveloped viruses generate a cytopathic effect in a range of amoebae and are released in amoebal EVs (Folkins et al., 2020; Dey et al., 2021). Hence, what roles may viruses play within amoebae (possibly in concert with internalized bacteria) regarding their expression of EVs and in modulating/avoiding human host immune responses? Important first insights to such mechanisms have been reported by Dey et al. (2022), who first described amoebic mitochondrial impacts by RNA viruses that appear to initiate and regulate apoptotic cell death. Overall this work points to research gaps that could be important to understand from an evolutionally perspective as well as to better describe human dose-response relationships for ARB pathogens.

Taking the above findings of NCLDV evolution, their likely (but not documented) presence within EVs and that specific genes from *Legionella* may be packaged within EVs – illustrates the is much to understand in the role that EVs may play in predator-prey interactions of ARB pathogens – and possibly much more to learn as to what actually leads to *Legionella* infection in human macrophages and alveolar epithelial cells.

## 3 Considerations for pro-active management of *L. pneumophila*

Managing emerging hazards has taken a more wholistic perspective in the last decade, and such an approach maybe particularly pertinent to legionellosis management. Stepping back to a broader view, we have an increasingly urbanized world population with its pollution becoming a dominant public health concern (via what is call the ‘pollutome’) (Pini et al., 2023). For chemical exposures there is also an increasing use of omics informed decisions about the exposome (Vlaanderen et al., 2021). Hence, it seems timely to also provide a One-Health lens to include microbial exosomes given their relevance to approximately one-third of community acquired (atypical) pneumonias (CAPs); which for those over 50 years of age is focused on healthcare settings, but recent work, reported for middle-eastern countries, has exposed CAP prevalent amongst young adults (20-40 years) within the broader community where legionellosis is also a major disease (Alhoufie et al., 2022).

As discussed above, molecular methods provide near real-time ways to target markers of pathogens, and in the current study the major vehicles for *L. pneumophila* amplification (FLA) and means for its dispersal and potential transmission (EVs). Clinical studies should also focus on the role amoeba trophozoites, EVs and fragments thereof may modulate human infection (including Pontiac fever), so such water monitoring targets could also be useful in clinical investigations. In the absence of being able to directly sample biofilm materials (such as though use of pipe wall coupons etc.), first flush tap samples are probably most informative of the last few meters where most problematic concentrations of ARB develop prior to aerosol exposures (Schoen and Ashbolt, 2011; Proctor et al., 2018). Hence, in concert with proactive management of flow conditions to keep cold waters below 25°C and hot water > 50°C, flushing of distal parts of water systems etc. (CDC, 2017), monitoring for amoebal aspects should provide the first hint of an increased potential for legionellae growth. Various qPCR protocols already exist for the major water-associated FLA and research should next focus on developing a more nuanced understanding of ARB growth conditions and targets.

## 4 Conclusions

While EVs seem to permeate all aspects of biology, surprisingly little is known about *L. pneumophila* and other amoeba-resisting pathogens’ role in driving EVs associated with FLA and engineered water systems. Seeking to provide proactive, rather than reactive management of legionellosis, by targeting management at the starting point for rapid amplification of *L. pneumophila*, i.e., a biomass of supportive amoebae, and promising EV telltales of their activity needs further examination. Molecular markers within EVs and of FLA would seem likely areas to first develop potential monitoring targets. Major data gaps identified during this study include: What role do internalized bacteria/viruses play in generating EVs and in modulating their behavior; Are amoebae induced to prey upon EVs; and, how FLA/EVs act in the gene pool leading to synchronized behavior within biofilm microbiomes and in human infection.

## Author contributions

One author contributed to the article and approved the submitted version.
